# Rare association of trisomy 13 with ectrodactyly and congenital diaphragmatic hernia

**DOI:** 10.1002/ccr3.4264

**Published:** 2021-05-25

**Authors:** Wafaa N. Al Rawi, Wala'a Al‐Safi, Ashraf N. Abuobayda, Nabil S. Elmansoury, Abhijeet S. Lonikar, Anas Alshorman, Hasansaheb D. Maldar

**Affiliations:** ^1^ Department of Pediatrics SSMC Hospital Abu Dhabi United Arab Emirates

## Abstract

Our findings expand the known clinical features of trisomy 13 by including ectrodactyly as a possible Trisomy 13‐associated limb malformation. We highlight the importance of performing antenatal genetic test to establish more specific treatment plan.

## Graphical abstract

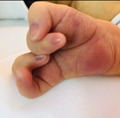

## 1 INTRODUCTION

In 2020, we reported one of the few cases of Trisomy 13 with ectrodactyly, a rare form of limb deformity. Herein, we report another newborn who suffered from Trisomy 13 and ectrodactyly. The patient also possessed another unusual manifestation of Trisomy 13, congenital diaphragmatic hernia, a life‐threatening pathology.

Trisomy 13, or Patau syndrome,^1^ is characterized by a wide range of anomalies, including complex heart and brain malformations. Resuscitation at birth is typically abandoned due to the poor prognosis of this syndrome and the associated lethal anomalies. One of the rare presentations of limb malformations in Trisomy 13 is ectrodactyly, a split‐hand or split‐foot malformation caused by an abnormal median cleft of the hand or foot, giving a lobster claw‐like appearance to the hand and/or foot.^2^ It was first described by Urioste et al.^3^ To the best of our knowledge, only three cases of ectrodactyly in patients with Trisomy 13, including the one previously published by our group, have been reported to date.^3^, ^4^, ^5^ Here, we report another case of ectrodactyly in a patient with Trisomy 13. Interestingly, the patient also presented with another infrequently described feature of Trisomy 13: congenital diaphragmatic hernia (CDH)^6^, ^7^. CDH is a serious malformation, which occurs due to a developmental defect in the diaphragm, allowing abdominal viscera to herniate into the chest and resulting in lung hypoplasia and pulmonary hypertension. Thus, CDH is associated with a significant risk of morbidity and mortality.

## 2 CASE REPORT

A male newborn weighing approximately 2400 g was delivered spontaneously at 36 weeks' gestation as the first child of nonconsanguineous parents. At 29 to 30 weeks' gestation, fetal ultrasonography detected left CDH; bilateral cleft lip and palate; and bilateral, echogenic, and enlarged kidneys. Parents declined amniocentesis. Immediately after delivery, the patient was intubated and ventilated. His Apgar score was 4 at 1 minute and 7 at 5 minutes.

**FIGURE 1:**
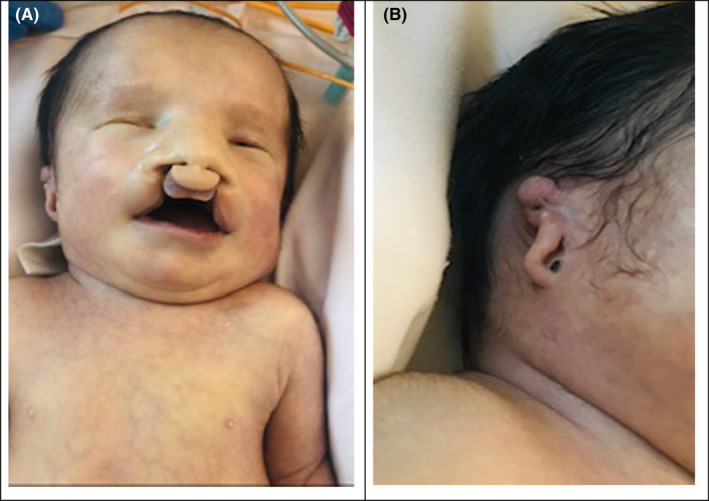
A, B, Bilateral microphthalmia, bilateral cleft lip and palate, and malformed and low‐set ears

**FIGURE 2:**
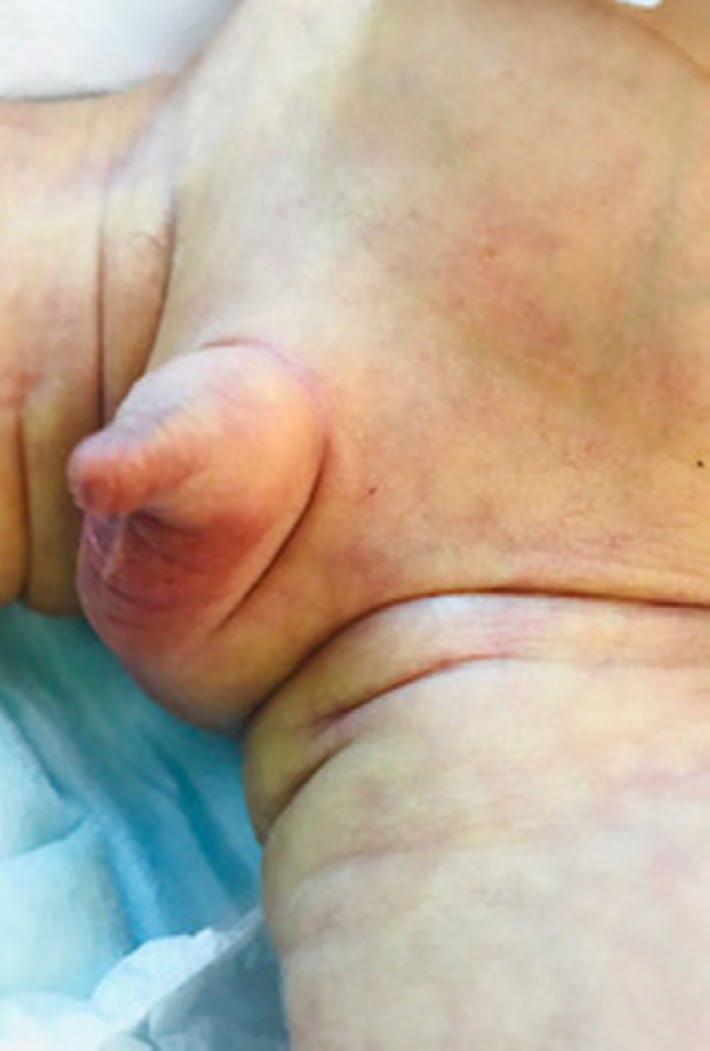
Bilateral cryptorchidism

**FIGURE 3:**
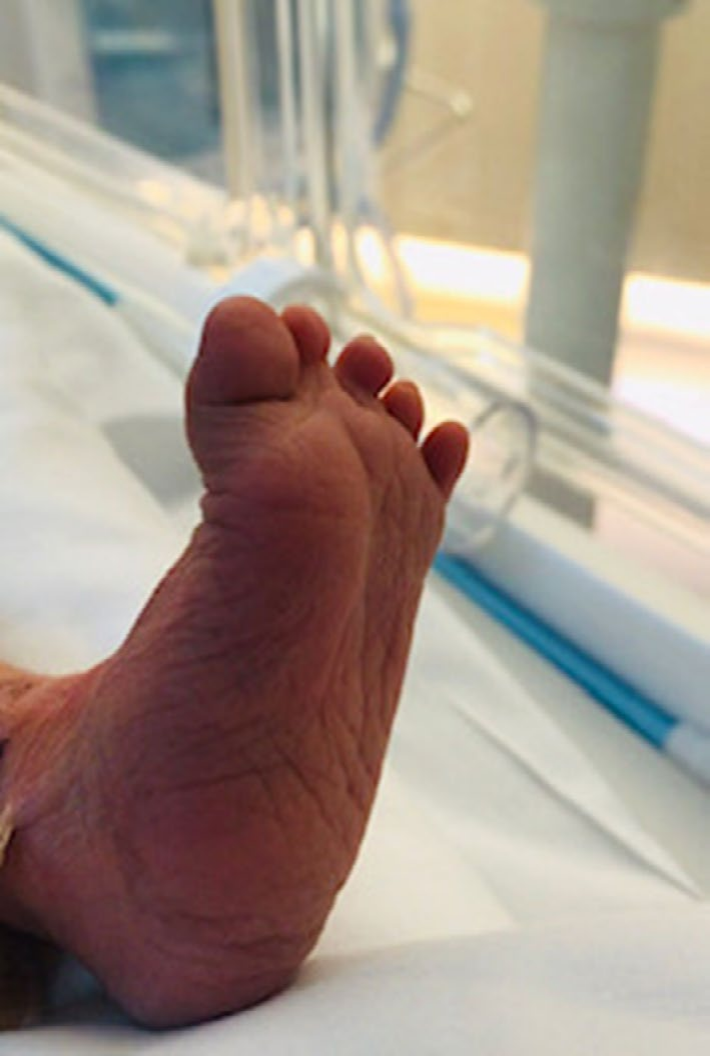
Rocker bottom feet

**FIGURE 4:**
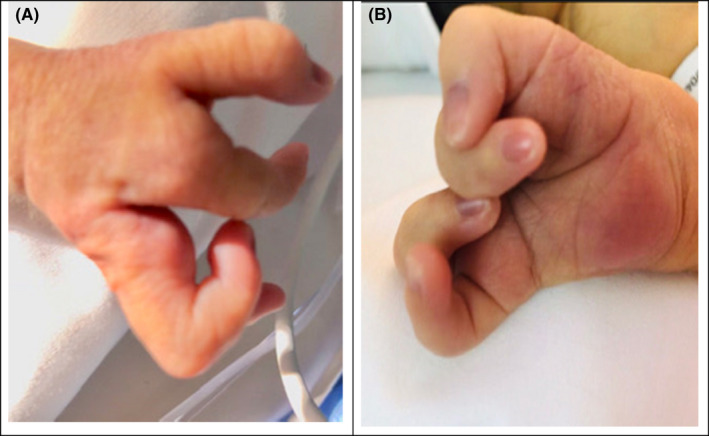
A, B, Right split lobster claw‐like hand, it has four fingers and deep medial cleft

Physical examination revealed multiple associated malformations, such as microphthalmia, bilateral cleft lip and palate, deformed left ear, scaphoid abdomen, micropenis, bilateral cryptorchidism, rocker‐bottom feet, right‐hand ectrodactyly with four fingers (oligodactyly), and deep medial cleft, giving the appearance of lobster claws (Figures 1, 2, 3, 4). Left CDH and bilateral hydroureter and hydronephrosis were confirmed by radiography. Brain ultrasonography results revealed cerebellar hypoplasia and hypogenesis of the corpus callosum, and echocardiography results showed mild tricuspid regurgitation and a deformed/calcified tricuspid valve. Trisomy was suspected, and the diagnosis of nondisjunction Trisomy 13 was confirmed by genetic testing (karyotype 47XY, +13).

The infant required high‐frequency oscillator ventilation and inhaled nitric oxide for severe respiratory failure, refractory hypoxia, and inotropic support. He deteriorated with no response and died on his fourth day of life.

## 3 DISCUSSION

**FIGURE 5:**
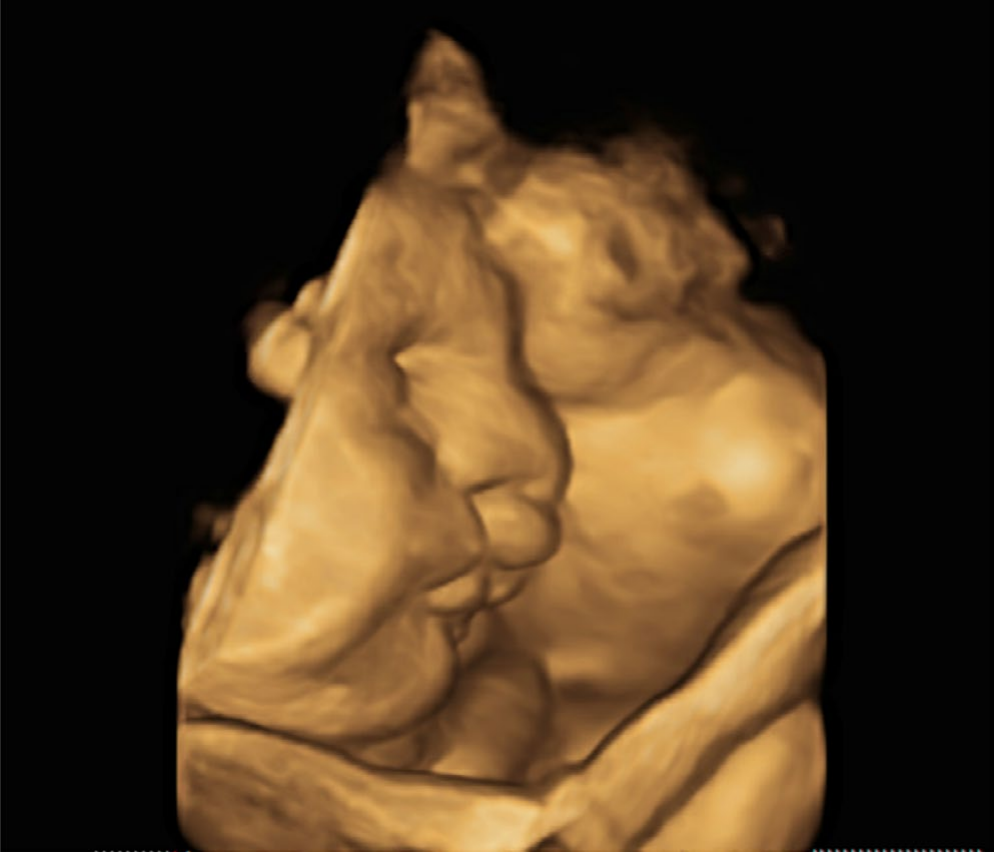
3D imaginary of face

**FIGURE 6:**
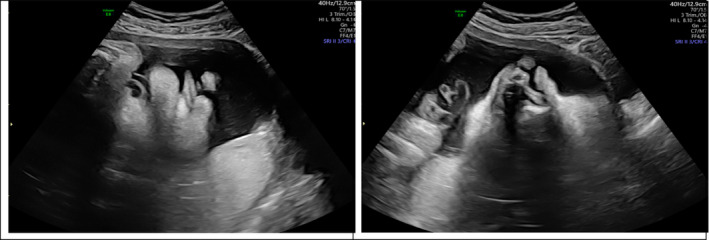
Bilateral facial cleft

**FIGURE 7:**
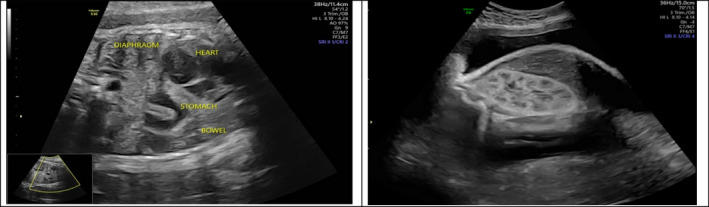
Stomach and bowel in chest‐CDH

**FIGURE 8:**
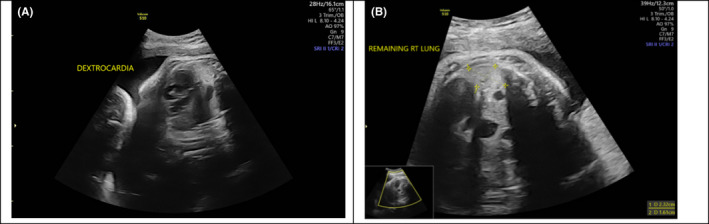
A, Heart pushed to the right side. B, Remaining of the right lung

**FIGURE 9:**
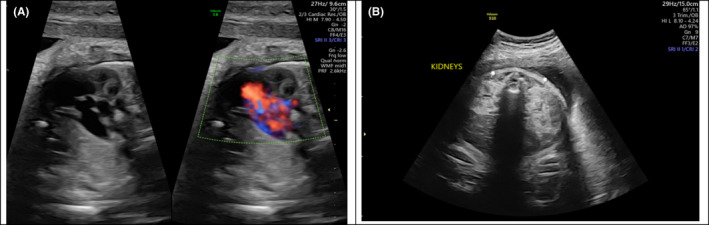
A, Heart with VSD. B, Bilateral echogenic kidneys

**FIGURE 10:**
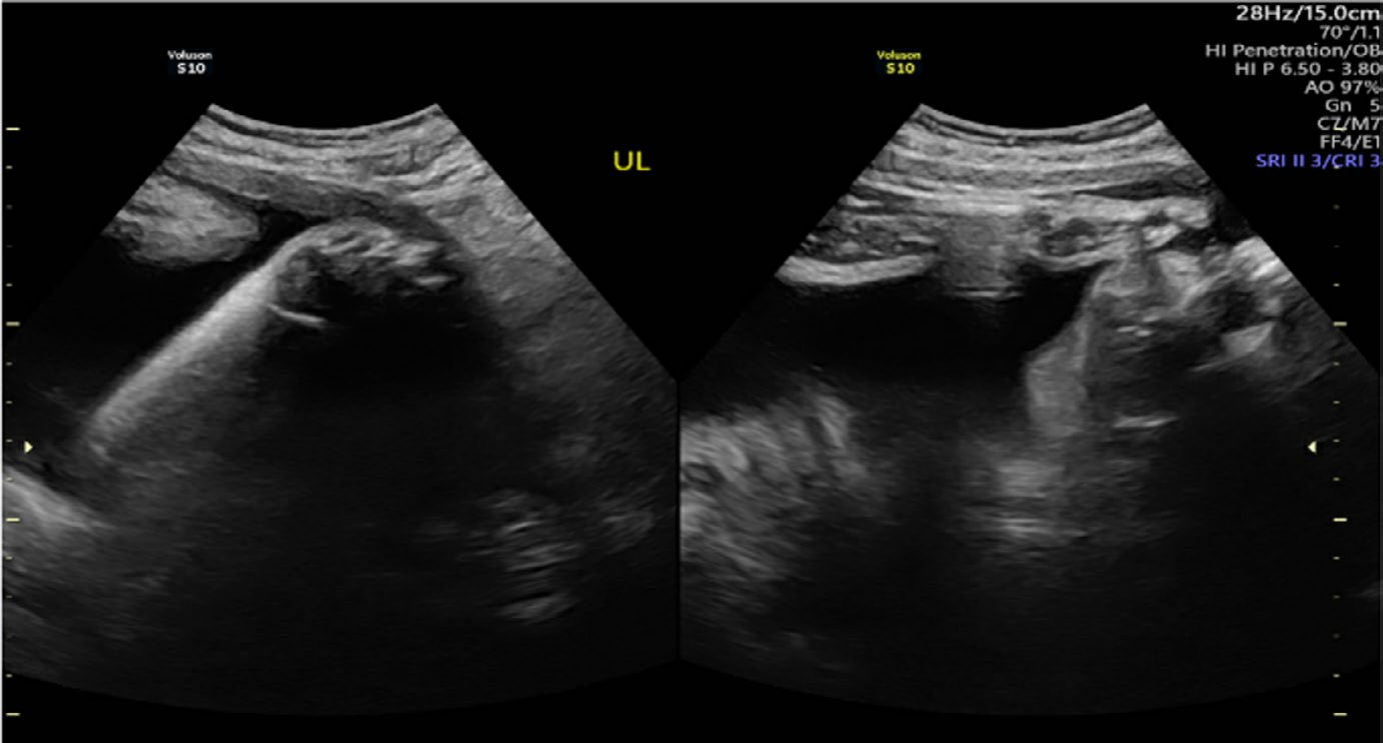
Clenched hand

Trisomy 13 is a common aneuploidy with a median survival of less than few months. Affected infants usually present with profound mental retardation, microcephaly, microphthalmia, cleft lip, cleft palate, hypertelorism, malformed ears, abnormalities of the central nervous system, renal malformations, congenital cardiac defects, capillary hemangiomas (most frequently on the center of the forehead), and limb malformation. Males may also be affected by cryptorchidism. In addition, limb malformations, including polydactyly, flexion and possible overlapping of fingers, and prominent heels of the feet, are common in infants with Trisomy 13 syndrome. However, ectrodactyly, a deformity affecting the central rays of the hands and/or feet and occurring as an isolated entity or as part of a syndrome, is a rare presentation in infants with Trisomy 13.^5^ Indeed, only a few reported cases have described such an association. Our patient also presented with CDH, another rare finding in Trisomy 13. CDH is a life‐threatening condition in infants and can be a major cause of death. It either can be an isolated anomaly or associated with other abnormalities, such as major structural malformations, chromosomal abnormalities, and/or single gene disorders. Severe anomalies associated with CDH may influence diagnosis, treatment, and survival. In our patient, an antenatal scan detected the presence of left CDH as an anomaly (Figures 5, 6, 7, 8, 9, 10). Although a prenatal genetic diagnosis of trisomy would have aided decision‐making and enabled the creation of a treatment plan, it was not performed due to lack of consent from the parents. Upon birth, the presence of such multiple congenital anomalies combined with CDH suggested a complex chromosomal diagnosis, which was confirmed by postnatal karyotyping as Trisomy 13. This result deferred the decision of surgical management, as the prognosis for CDH is poor in the presence of an abnormal chromosomal microarray case.

## 4 CONCLUSION

This case study reports a rare case of a patient with Trisomy 13 that presented both ectrodactyly and CDH, which are very rarely presented with Trisomy 13. Our findings expand the known clinical features of Trisomy 13 by including ectrodactyly as a possible Trisomy 13‐associated limb malformation. In addition, CDH is an unusual Trisomy 13 manifestation for which creating a treatment plan is difficult without a confirmed diagnosis. Thus, we highlight the importance of performing antenatal genetic tests to establish more specific treatment strategies and offering genetic counseling and parental support for cases in which multiple congenital defects have been detected *in* *utero*.

## CONFLICT OF INTEREST

None declared.

## AUTHOR CONTRIBUTIONS

All authors shared the writing, reviewed the results and approved the final version of the manuscript.

## ETHICAL APPROVAL

We confirm that the manuscript has been read and approved by all named authors. The protection of intellectual property associated with this manuscript had been in our consideration.

## Data Availability

The data that support the findings of this study are available from the corresponding author, upon reasonable request.
